# A clinicopathological review of 33 patients with vulvar melanoma identifies c-KIT as a prognostic marker

**DOI:** 10.3892/ijmm.2014.1659

**Published:** 2014-02-14

**Authors:** VIOLA A. HEINZELMANN-SCHWARZ, SHERI NIXDORF, MEHRNAZ VALADAN, MONICA DICZBALIS, JAKE OLIVIER, GEOFF OTTON, ANDRÉ FEDIER, NEVILLE F. HACKER, JAMES P. SCURRY

**Affiliations:** 1 Ovarian Cancer Group, Lowy Cancer Research Centre, School of Women’s and Children’s Health and Prince of Wales Clinical School, University of New South Wales, Sydney, NSW 2052, Australia; 2 Gynaecological Cancer Centre, Royal Hospital for Women, School of Women’s and Children’s Health, University of New South Wales, Sydney, NSW 2031, Australia; 3 Gynecological Research Group, Department of Medicine, University Hospital Basel, University of Basel, Switzerland; 4 School of Medicine, Tehran University of Medical Sciences, Tehran, Iran; 5 Hunter Area Pathology Service and University of Newcastle, John Hunter Hospital, Newcastle, NSW 2310, Australia; 6 School of Mathematics and Statistics, University of New South Wales, Sydney, NSW 2052, Australia; 7 Hunter Centre for Gynaecological Cancer, John Hunter Hospital, Newcastle, NSW 2310, Australia

**Keywords:** vulvar melanoma, prognostic predictors, risk factors, c-KIT, lichen sclerosus

## Abstract

Vulvar melanoma is the second most common vulvar cancer. Patients with vulvar melanoma usually present with the disease at a late stage and have a poor prognosis. The prognostic predictors reported in the literature are not unequivocal and the role of lichen sclerosus and c-KIT mutations in the aetiology of vulvar melanoma is unclear. Breslow staging currently seems to be the most adequate predictor of prognosis. We thus performed a clinicopathological and literature review to identify suitable predictors of prognosis and survival and investigated the expression of c-KIT (by immunohistochemistry) in patients with vulvar melanoma (n=33) from the Gynaecological Cancer Centres of the Royal Hospital for Women (Sydney, Australia) and John Hunter Hospital (Newcastle, Australia). Our series of 33 patients fitted the expected clinical profile of older women: delayed presentation, high stage, limited response to treatment and poor prognosis. We identified 3 patients (9.1%) with lichen sclerosus associated with melanoma *in situ*, although no lichen sclerosus was found in the areas of invasive melanoma. No patient had vulvar nevi. We identified a) Breslow’s depth, b) an absence of any of the pathological risk factors, such as satellitosis, in-transit metastasis, lymphovascular space invasion (LVSI) and dermal mitosis, c) removal of inguino-femoral lymph nodes, d) lateral margin of >1 cm, and e) c-KIT expression as valuable prognostic predictors for disease-free survival. We conclude that c-KIT expression is, apart from Breslow’s depth, another valuable predictor of prognosis and survival. Lichen sclerosus may be associated with vulvar melanoma.

## Introduction

Vulvar melanoma is the second most common vulvar cancer with an incidence of 0.1 per 100,000 individuals, presenting typically in post-menopausal women (1,2). Patients with vulvar melanoma, due to the lack of body awareness, false modesty and neglect, usually present with the disease at a late stage and have a poor overall prognosis, with reported 5-year survival rates ranging from 8 to 61% (3–10). This is unlike cutaneous melanomas where, as a result of increased public and clinical awareness, many patients are now diagnosed at an early stage.

The aetiology of vulvar melanoma is poorly understood but is believed to arise *de novo*, that is, to develop from the malignant transformation of a single junctional melanocyte *in situ* (4). A genetic basis is suspected, which may explain why caucasions have a 3-fold higher incidence rate than individuals of African descent (11) and why individuals of African descent have a 2-fold shorter median survival than caucasions (12). Activating c-KIT mutations have been found in patients with vulvar melanoma (13,14). By contrast, as previously demonstrated, gene mutations for cutaneous melanomas were irrelevant in vulvar melanomas (BRAF, NRAS), indicating that these two diseases have a different origin (13,15).

Lichen sclerosus (LS) is also suspected to cause vulvar melanoma, and LS has indeed been reported with vulvar melanoma in 6 cases (17–20). Whereas 5 childhood vulvar melanomas cases have been reported, only 1 among thousands of adult vulvar melanoma patients have been reported (18). The mechanisms involved, however, are not clear. LS, itself an inflammatory dermatosis of unknown origin presenting with whitening of the skin and pruritus (21), is the most common precursor of HPV-negative squamous cell carcinoma of the vulva, where the postulated pathogenesis is a ‘scar cancer’ similar to Margolin’s ulcer (22,23). The difficulty of distinguishing vulvar melanoma in the setting of LS from benign pigmented lesions of the genitals is acknowledged (24).

There is no consensus regarding the adequate staging system and the treatment for vulvar melanomas. The standard FIGO staging, as used for squamous cell carcinoma, is not satisfactory. In vulvar melanomas, lesions are usually much smaller and prognosis is related to depth rather than diameter. For this reason, Breslow staging, which takes into account the depth of tumour rather than its size, seems to be most adequate and until today the best predictor of prognosis (25–29). This was also confirmed by a recent American study with 85 cases of melanomas of the female genitalia (30): a higher Breslow depth was associated with declining survival, whereas other histopathological features, such as ulcerations, increasing mitotic index and the presence of atypical melanocytic hyperplasia were not associated with a significant survival difference. Three basic histotypes (superficial spreading melanoma, mucosal lentiginous melanoma and nodular melanoma) have been described with varying incidence rates (5,6,9,31).

It is an ongoing search for reliable histological features that allow the prognosis of vulvar melanoma; however, the majority of studies (small case series and retrospective reviews) have delivered inconclusive results. Thus, we performed a) a comprehensive literature review, b) a clinicopathological review of 33 vulvar melanoma cases of an Australian cohort to identify potential histopathological predictors of outcome, and c) immunohistochemistry for c-KIT expression in a respective tissue microarray.

## Patients and methods

### Comprehensive literature review

The systematic literature review was performed using the online websites, PubMed, Medline and Cochrane for the key words ‘vulva melanoma’, ‘mucosal melanoma’, ‘melanoma’, ‘melanomas’, ‘vulvar’, ‘vulva’ and ‘vulvar neoplasm’. The retrieval was limited from 1990 to 2012 and included epidemiological studies, literature reviews, retrospective series, prospective series, meta-analyses and molecular analyses. Studies with <10 patients were excluded, as were individual case reports. Studies were reported as to their year of publication, the number of enrolled patients, type of study, mean age of patients, 5-year overall survival, and study results and clinicopathological predictors of outcome (Table I).

### Clinicopathological review of our study cohort

Upon obtaining ethical approval and informed consent, we identified and enrolled 33 patients with vulvar melanoma at the Gynaecological Cancer Centre of the Royal Hospital for Women, Sydney (20 patients, incepted 1987) and the Gynaecological Cancer Centre of John Hunter Hospital, Newcastle (13 patients, incepted 1991). The following information was retrieved from the charts of the patients: age (diagnosis/menopause/relapse/death), duration of symptoms (months), menopausal status, family history of melanoma, location of melanoma, mode of detection, palpable groin nodes, lymph nodes removed/positive, CT scan results, Breslow’s depth, type of surgery, chemotherapy/immunotherapy/radiotherapy (number of sessions and dose), treatment side-effects, site/location of recurrence, cause of death, and relapse-free and overall survival.

Characteristics which were assessed included diagnosis, pathogenic type (superficial spreading, mucosal lentiginous melanoma, other), predominant cell type (epithelioid, spindle or other), ulcerations, Breslow’s depth, tumour infiltrating lymphocytes (TILS), regression (dermal fibrosis, lymphocytic infiltrate and, in cases of pigmented melanomas, melanophages), lymphovascular space invasion (LVSI), satellitosis (discrete tumour nests >0.05 mm in diameter, separated from invasive tumour by ≥0.3 mm) and in-transit metastases (>20 mm from invasive tumour), margins (involved by *in situ* or invasive melanoma), adjacent abnormal melanocytic proliferation and LS.

### c-KIT immunohistochemistry of tissue microarrays

An independent, blinded pathological review of all haematoxylin and eosin slides was performed by a pathologist specialised in vulvar pathology (Dr J.P. Scurry). These slides where marked for vulvar melanoma and two 1-mm cores were transferred onto a tissue microarray, using the ATA-100 Advancer Tissue Arrayer (Chemicon International, Temecula, CA, USA). Cores also included control tissues from negative inguinal lymph nodes that were surgically sampled. Immunohistochemistry was performed in the Bond™-X System (Leica Biosystems, Wetzlar, Germany) using the polyclonal rabbit Anti-human CD117 antibody (c-KIT; Dako, Carpinteria, CA, USA) at a 1:400 dilution followed by secondary detection with the Bond™ Polymer Refine Detection kit combining anti-mouse and anti-rabbit antibodies (Leica Biosystems). Prior to staining, antigen retrieval was performed at 95°C for 15 min in the PT Link (Dako) using the EnVision™ FLEX target retrieval solution, low pH (50×; Dako), followed by a water wash. Evaluation of the intensity of c-KIT cytoplasmic and membrane protein expression was performed by two researchers independently and consensus was reached. For the purpose of this analysis either cytoplasmic or membrane staining of 3+ intensity was taken as strong c-KIT expression.

### Statistical analysis

The clinicopathological data were collected in an in-house research database based on ACCESS (Microsoft Windows, USA) and analysed with SAS statistical software (SAS Institute Inc., Cary, NC, USA). Mean values with standard deviation and range were generated for longitudinal datasets and nominal data were presented as percentages. Potential risk factors for relapse and mortality were assessed through Kaplan-Meier curves and Cox proportional hazards models. As the number of cases was limited, the significance of each hazard ratio (HR) was primarily assessed by their effect size as p-values alone were likely to miss important results.

## Results

### Comprehensive literature review: predictors of outcome and molecular targets for vulvar melanoma

Our literature review revealed 46 studies with >10 patients enrolled with vulvar melanoma (Table I). These studies often combined both mucosal (including those of the vulva) and cutaneous melanomas. Out of these 46 studies, 23 were retrospective studies, with 48.9% comprising the majority of publications in vulvar melanoma research, 8 studies were literature reviews, 11 comprised analyses of molecular targets, and 1 was a meta-analysis. No Cochrane review has been performed to date.

The unequivocal clinical predictors of patient outcome that were identified were inguinal lymph node status (either via sentinel or standard lymphadenectomy; 11 studies) and Breslow’s depth (9 studies). Ambiguous clinical predictors included tumour ulceration (4 studies), age at diagnosis (3 studies) and DNA ploidy (3 studies).

Molecular targets suspected to be relevant in mucosal melanomas have been investigated in 11 studies. Mutations in mucosal melanomas were found in *p53* (3 studies), in *c-KIT* (2 studies) and in key kinases of the PI3K/AKT/mTOR- (1 study) and RAS/RAF/MEK/ERK-pathways (1 study). Mutations in *BRAF* or *NRAS* in mucosal melanomas were not found (1 study each) and evidence of the involvement of viral infections (HPV, HSV, polyomaviruses) in vulvar melanoma was not found either. Notably, high-throughput transcription profiling experiments on vulvar melanomas have not been performed to date.

### Clinicopathological and immunological characteristics of our cohort

The clinicopathological and immunological characteristics of the 33 patients of our cohort are summarized in Table II. The mean age at diagnosis was 67.5 years (range, 34–95 years) and was higher compared to that of the 47 literature review studies (62.2 years; range, 53–80 years). Patients presented with symptoms for an average of 28.2 months (range, 2–112 months) and in 72.2% of the cases detected the lesion themselves. By virtue of the advanced mean age at diagnosis almost three quarters (73.5%) of our patients were post-menopausal. The vast majority of the patients (93.8%) did not have a family history of melanoma. The most common location of vulvar melanomas was at the labia minora (31.6%) and was multifocal (26.3%).

The majority of patients presented with an advanced Breslow stage of V (56.3%) and had a mean tumour size of 21.9 mm (range, 5–50 mm), deep margin of 5.20 mm (range, 0–22 mm) and lateral margin of 3.9 mm (range, 0–12 mm). Most of our patients received radical local excision. Of the 4 patients that had received a radical vulvectomy, 3 had multifocal disease and the other was treated by an outside consultant. The majority of the patients (68.8%) underwent at least an unilateral inguino-femoral lymphadenectomy without any notable side-effects (in particular no lymphoceles or lymphoedema). For the majority of patients, this was the only adjuvant treatment received: only 25% received chemotherapy, 18.2% immunotherapy and 38.9% radiotherapy. Seventy percent of the patients relapsed, with local and distant metastases equally common: the most common local recurrence was at the vulva (30.8%). The median time to relapse was 40 months and to death 44 months. Fifty-five percent of the patients succumbed to the disease, mostly due to causes related to their disease (90%).

**Figure 1:**
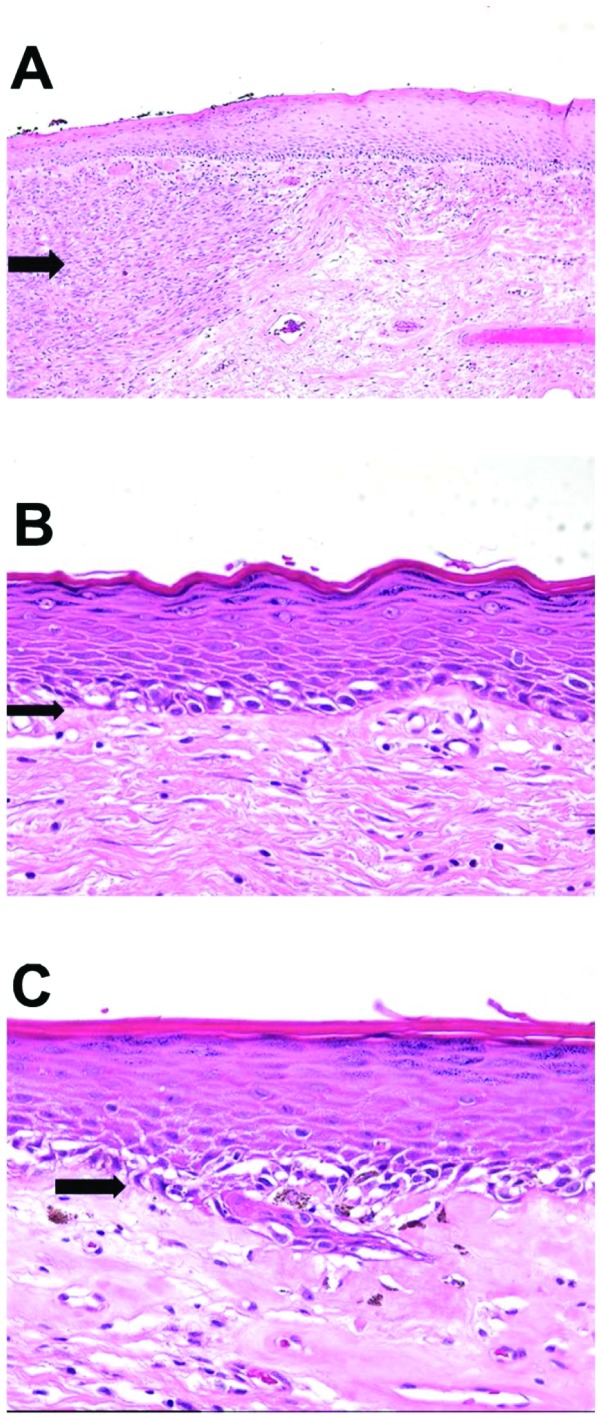
(A) Spindle cell melanoma (arrow) with fibrosis adjacent to the melanoma. (B) Next to the invasive melanoma, there is melanoma *in situ* (arrow) associated with fibrosis. (C) Further away from the melanoma, there is melanoma *in situ* (arrow) and lichen sclerosus with characteristic dermal hyalinisation of lichen sclerosus.

**Figure 2:**
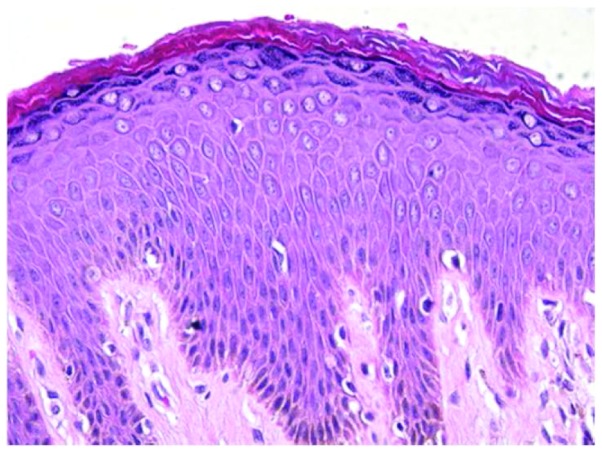
Scattered large atypical melanocytes without melanocytic proliferation observed at the periphery of a vulvar melanoma.

**Figure 3:**
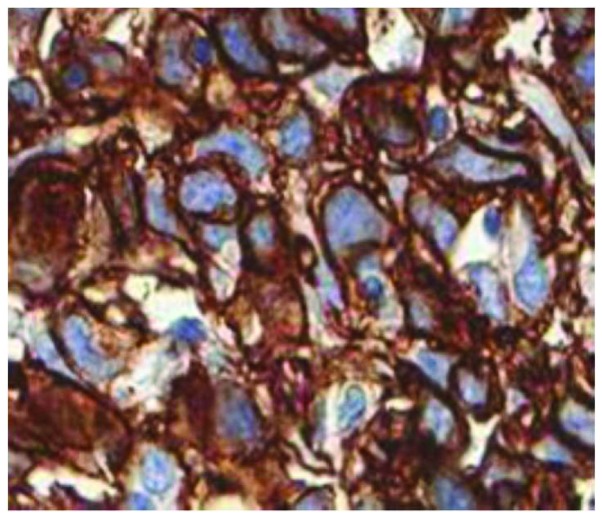
Strong c-KIT expression in invasive melanoma of the vulva.

The majority of patients presented with a clinically or pathologically detected ulceration (53.3% or 88.9%, respectively) of a large tumour nodule of spindle cell type (52.9%), with a mean of 7.3 dermal mitoses per mm^2^ (range, 1–40) and high TILS (58.8%). The majority of the patients did not have regression (66.5%), satellitosis (88.9%), in-transit metastases (83.3%), LVSI (88.9%), or LS (88.9%). In our cohort, 3 cases of LS with vulvar melanoma were identified (Table III). In all these patients, LS was observed with or without melanoma *in situ*, but always disappeared beneath the invasive melanoma. No pre-existing nevi were found, but 2 patients showed large single melanocytes at the edge of the melanoma *in situ*. Representative macroscopic images of a vulvar melanoma specimen and histological examples for various pathological features are presented in Figs. 1 and 2. An example of a strong c-KIT expression in an invasive melanoma of the vulva is illustrated in Fig. 3.

### Predictors of outcome for vulvar melanoma identified in our cohort

**Figure 4:**
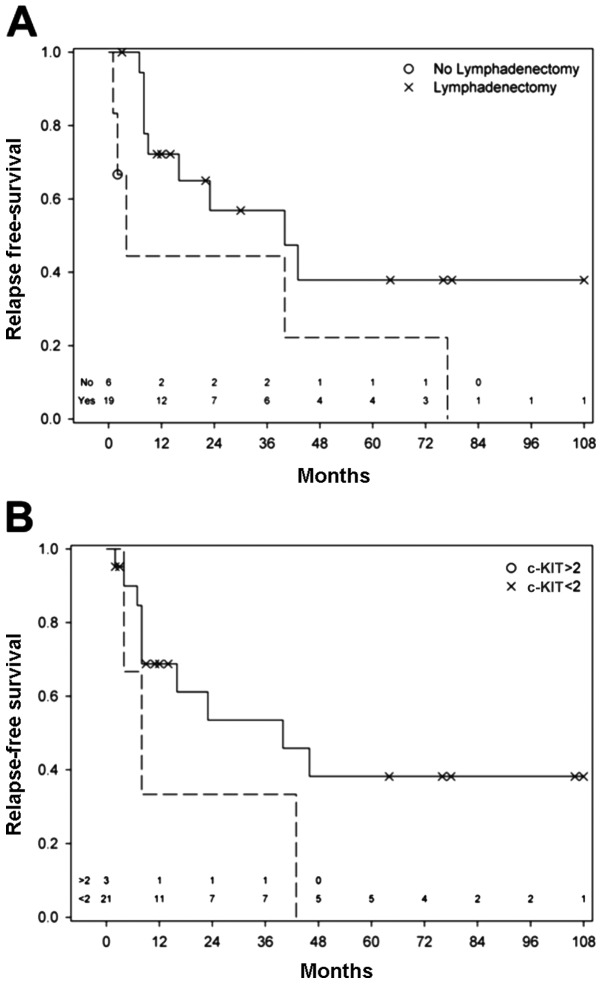
Kaplan-Meier survival curves for relapse-free survival over a specific time frame (months) for patients (A) who had lymphadenectomy performed (x) versus the ones who had not (o); and (B) for patients whose tumours expressed strong c-KIT expression (o) versus those whose tumours did not (x).

Our study confirmed the known predictive clinicopathological characteristics Breslow’s depth [relapse-free survival (RFS): HR=1.08, p=0.049] and lymphadenectomy (RFS: HR=0.376, p=0.087, Table IV and Fig. 4A). These were particularly important in relation to recurrence. No significant results for positivity of lymph nodes were found in our series, possibly due to the low numbers of positive lymph nodes. In the presence of a lateral margin of >10 mm [disease-free survival (DFS): HR=2.7, p=0.21] and a strong (intensity 3+) c-KIT expression (DFS: HR=1.8, p=0.49; RFS: HR=3.13, p=0.108; Fig. 3), Breslow’s depth becomes less important as regards the outcome (Table IV, Fig. 4B).

**Figure 5:**
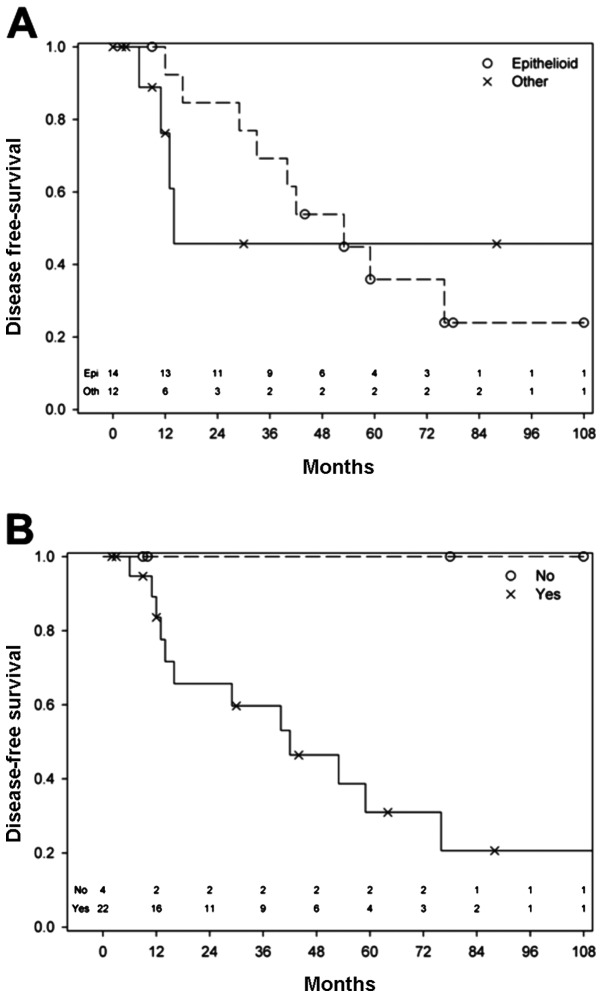
Kaplan-Meier survival curves for disease-free survival over a specific time frame (months) for patients (A) with epithelioid or mixed epithelioid tumours (o) or other histotypes (x); and (B) which expressed at least one of the following suspicious pathological features: satellitosis, in-transit metastases, lymphovascular space invasion (LVSI) or dermal mitosis (x) versus the patients which did not (o).

The presence of epithelioid cells within a vulvar melanoma, even when mixed in combination with spindle or nodular cells, predicted a better prognosis for these patients (HR=0.82, p=0.75) (Table IV, Fig. 5A). Of the 3 patients, who contribute to the plateau in the non-epithelioid curve in the Kaplan-Meier plot, 2 had a spindle/epithelioid and the other had another cell type, thus supporting our findings.

Due to our cohort size and the low numbers of certain pathological characteristics, we looked in a combined approach at high-risk pathological features, such as satellitosis, in-transit metastases, LVSI and dermal mitosis. This meant that the presence of any of these 4 features within a cancer was taken for the purpose of this analysis as the presence of a high-risk pathological feature. Using this approach, we found that in the absence of at least one of these features, none of the patients died (log-rank test, p=0.088), which had a sensitivity of 100% (Fig. 5B). As regards recurrence, the presence of at least one of these pathological features increased the risk of recurrence from the disease by a factor of 5 (HR=5.02).

### Independent predictors of outcome and c-KIT expression

We modelled the identified predictors of prognosis with each other in order to identify the degree of correlation between the predictive parameters. In all models, the strongest predictors for outcome, both in respect of relapse and survival, was the absence of any of the pathological high-risk characteristics, such as satellitosis, in-transit metastases, LVSI or dermal mitosis.

In combination with the pathological high-risk characteristics, the strongest predictors for earlier relapse were c-KIT expression [adjusted HR (aHR)=2.51, Table IVA] and Breslow’s depth (aHR=1.12, Table IVA). With the increasing depth of the melanoma, lymphadenectomy presents with a HR of 6.86 (p=0.011) and Breslow’s depth remains statistically significant (HR=1.13, p=0.0079). Breslow’s depth, in the presence of some of the other factors, seems to be more important for recurrence than DFS. None of the classical adjuvant treatment options, including immunotherapy showed any benefit in our study.

When modelled together, the strongest predictors of earlier death were pathologically high-risk characteristics, followed by lateral margin (>10 mm, aHR=7.32, Table IVB), a strong c-KIT expression (aHR=3.34, Table IVB) and lymphadenectomy (aHR=0.3, Table IVB). For DFS, Breslow’s depth loses its strong predictive value when compared to a lateral margin of >10 mm and a strong c-KIT expression. The comparison of the lateral margin to strong c-KIT expression identified the lateral margin as more important for survival.

The combined multivariable model for the prediction of DFS consisted of a) lymphadenectomy, b) absence of any of the pathological high-risk characteristics, c) strong c-KIT expression, and d) Breslow’s depth, and was highly statistically significant (p=0.0004).

## Discussion

Whilst in recent years great achievements in disease awareness, in early diagnosis, and in the treatment of cutaneous melanomas with subsequent benefits in morbidity and mortality have been made, no similar development exists for vulvar melanomas. Patients with vulvar melanomas usually present with the disease at a late stage and have a poor prognosis. Its aetiology is poorly understood and the prognostic predictors reported in the literature are not fully conclusive. Research into vulvar melanoma is also limited due to the low incidence of cases per centre and low numbers of international collaborative studies or meta-analyses.

Our comprehensive literature review of 46 studies published from 1990 until 2012 identified Breslow’s depth and the inguinal lymph node status as unequivocal and tumour ulceration, age at diagnosis, and DNA ploidy as less clear or ambiguous clinical predictors of outcome. On the molecular/genetic level, mutations in *p53, c-KIT* and kinases of the PI3K/AKT/mTOR- and RAS/RAF/MEK/ERK-pathways have been reported in association with vulvar melanoma. p53 is a tumour suppressor gene (65), c-KIT is a receptor tyrosine kinase, mutations of which are integral for tumour growth and progression (66), and PI3K/AKT/mTOR- and RAS/RAF/MEK/ERK-pathways regulate growth and proliferation (67,68). By contrast, neither mutations in *BRAF* or *NRAS* nor an involvement of viral infection were found. These data may not be conclusive and high-throughput transcription profiling experiments on vulvar melanomas are likely to identify additional genes, the mutations of which are associated with vulvar melanoma.

In our cohort of 33 adult patients, 3 cases (9.1%) of vulvar melanoma with LS (Table III) were identified, suggesting an association. This is noteworthy, as to date, reported cases of LS associated with vulvar melanomas were mainly limited to juvenile cases (16–20) (Table III). In our 3 cases, the LS was present in melanoma *in situ*, but disappeared in the invasive melanoma, where dermal hyalinisation was replaced by desmoplasia. The limited number of reports on the association of LS with adult vulvar melanoma may be due to under-reporting and lack of recognition.

In our cohort, we also found an increased c-KIT protein expression in approximately half of the patients, suggesting a role of c-KIT in vulvar melanoma. In fact, c-KIT mutations have been shown to be more common in vulvar than cutaneous melanomas (13,14). c-KIT is a receptor tyrosine kinase regulating a variety of biological responses, such as chemotaxis, cell proliferation, apoptosis and adhesion in many cell types, including melanocytes, and activating KIT mutations are integral for tumour growth and progression (69); however, their role in vulvar melanoma is yet not known.

Over the years, a number of histopatological features have been shown to correlate with adverse prognosis. These include Breslow’s depth, ulceration, epithelioid cell type, microsatellitosis, regression, angiolymphatic involvement, high mitotic rate, amelanosis and association with an existing nevus (3,5,35,51). An American study demonstrated that increasing Breslow depth was associated with declining survival, whereas other histopathological features, such as ulceration, increasing mitotic index, and the presence of atypical melanocytic hyperplasia were not associated with a significant difference in survival (30). A recent Chinese study revealed that macroscopic tumour growth and treatment method were independent prognostic factors for overall survival (70). Our study confirmed Breslow’s depth and lymphadenectomy as strong predictors for recurrence and poorer DFS.

Our study also identified other predictive features. Among those was the absence of any of the pathological high-risk characteristics (satellitosis, in-transit metastases, LVSI or dermal mitosis) identified in a subset of patients with vulvar melanoma. These patients survived disease with a prediction of 100% sensitivity, making the absence of these characteristics strong predictors for outcome, both in terms of relapse and survival. This group of patients may qualify for follow-up after surgery, particularly when an optimal adjuvant therapy is not available. An increased c-KIT expression was also identified as a strong negative predictor of DFS and a strong positive predictor of earlier relapse. By contrast, no significant results for positivity of lymph nodes were observed in our study, possibly due to the low numbers of positive lymph nodes.

The identification of mutated genes, such as *c-KIT* and *p53* or increased levels of c-KIT in vulvar melanomas seems consistent with the current consensus that vulvar melanomas arise *de novo* from the malignant transformation of a single junctional melanocyte *in situ* (4). Indeed, we found single large junctional melanocytes adjacent to melanoma *in situ*, which has, to our knowledge, not been reported previously. Mucosal melanomas arise from an epithelium normally devoid of melanocytes; the significance of melanocytes in a location where they are not normally present therefore requires further investigation.

The treatment of vulvar melanomas has thus far been largely restricted to surgical options, with little prospective data and no randomised studies available. Following on the trend from cutaneous melanomas, the surgical approach for vulvar melanomas has changed from extensive to more limited procedures due to the recognition that no improvement in overall survival can be achieved with aggressive surgery despite increasing patient morbidity (32,71), and no benefit is found from pelvic lymphadenectomies in the absence of groin node metastases (72,73), similar to squamous cell carcinomas of the vulva. In the absence of adequate randomized controlled trials, adjuvant treatments included radiotherapy, chemotherapy, immunotherapy, and in one case targeted therapy. Immunotherapy using interferon α2b has shown significantly improved DFS in randomized controlled trials, but there is significant morbidity (74–76). The role of adjuvant radiotherapy is unknown and may only be used in the case of close surgical margins, whilst recurrent cancer in the absence of metastatic disease is best managed surgically.

An important development is the evidence that mucosal (vulvar melanomas are classified as mucosal) and cutaneous melanomas are distinct genetic entities and should be studied and treated as such (13,15,77). Gene mutations for cutaneous melanomas did not prove to be of relevance in vulvar melanomas (BRAF, NRAS) whilst p53 and c-KIT mutations were identified and may enable therapeutic options in the future. Pathological classifiers, such as satellitosis, in-transit metastases, LVSI and dermal mitosis can stratify patients who would profit from the investigation into c-KIT expression and the subsequent imatinib treatment. Imatinib is a targeted oral therapeutic agent against cutaneous melanomas. An Australian study has shown some efficacy with the treatment of imatinib in mucosal melanomas, including vulvar melanomas (78). More studies into the genetic background, making use of high-throughput transcription profiling technology increasingly becoming available, are required to develop targeted treatment options, particularly in high-risk groups.

International trials with imatinib or any other therapeutic option available in the future in high-risk vulvar melanomas will be beneficial, but will face all the difficulties associated with targeting very rare tumours. The centralization of care for patients with vulvar melanoma is inevitable. Whilst the surgical part of their treatment is best performed in a gynaecological cancer centre, ongoing care should best be shared within a multi-disciplinary approach, involving both gynaecological oncologists and melanoma centres.

## Figures and Tables

**Table I:** Literature review.

Author/(Refs.)	Year	Vulvar melanoma patients (n)	Melanoma location	Study type	Mean patient age (years)	5-year OS (%)	Results and predictors of outcome
Bradgate *et al* (3)	1990	50	Vulva	Retrospective series	N/A	35	Clinical stage, patient age, ulceration, cell type, mitotic rate
Trimble *et al* (32)	1992	80	Vulva	Retrospective series	58.5	60	Extent of vulva surgery not relevant
Tasseron *et al* (33)	1992	30	Vulva	Retrospective series	63.8	56	Ulceration
Piura *et al* (34)	1992	18	Vulva	Retrospective series	N/A	28.6	Positive inguinal lymph nodes not relevant
Ragnarsson-Olding *et al* (35)	1993	219	Vulva and other mucosal melanoma	Epidemiological study	75	35	Decrease in age-standardized incidence in Sweden
Look *et al* (36)	1993	16	Vulva	Retrospective series	59	N/A	Depth >1.75 mm predicts recurrence within 24 months
Phillips *et al* (37)	1994	71	Vulva	Prospective series	71–80	43.9	AJCC staging, Breslow’s depth
Dunton *et al* (38)	1995	N/A	N/A	Literature review	N/A	N/A	Breslow’s depth, lymph node dissection
Scheistroen *et al* (31)	1995	75	Vulva	Retrospective series	67	46	Tumour localization clitoris, DNA ploidy, positive inguinal lymph nodes
Raber *et al* (39)	1996	89	Vulva	Retrospective series	55.4	36.7	Breslow’s depth, Clark’s level, lymph node status
Trimble (40)	1996	N/A	N/A	Literature review	N/A	N/A	Breslow’s depth
Scheistroen *et al* (41)	1996	43	Vulva	Retrospective series	64	63	Angioinvasion, DNA ploidy
Strauss (42)	1997	N/A	Melanomas	Molecular analysis	N/A	N/A	p53 mutations
Jiveskog *et al* (15)	1998	N/A	Cutaneous vs. mucosal melanomas	Molecular analysis	N/A	N/A	NRAS mutations not relevant
De Matos *et al* (43)	1998	30	Vulva	Retrospective series	66	59	Regional metastases
Ragnarsson-Olding *et al* (5)	1999	219	Vulva	Epidemiological study	N/A	47	Breslow’s depth, ulceration, amelanosis
Larsson *et al* (44)	1999	19	Vulva	Retrospective series	N/A	23	Stage
Creasman *et al* (45)	1999	569	Vulva	Retrospective series	66	62	AJCC stage
Raspagliesi *et al* (46)	2000	40	Vulva	Retrospective series	58	48	Positive inguinal lymph nodes
Verschraegen *et al* (9)	2001	51	Vulva	Retrospective series	54	27	AJCC stage, Breslow’s depth
Irvin *et al* (8)	2001	14	Vulva	Retrospective series	58	42	Margins, inguinal lymphadenectomy
Ragnarsson-Olding *et al* (47)	2002	22	Vulva	Molecular analysis			p53 mutations
De Hullu *et al* (48)	2002	33	Vulva	Retrospective series	69	52	Sentinel lymphadenectomy
Finan and Barre (49)	2003	N/A	N/A	Literature review	N/A	N/A	Age, AJCC stage
Ragnarsson-Olding (2)	2004	1,442	Vulva	Meta-analysis	N/A	N/A	Breslow’s depth, ulceration, amelanosis, angioinvasion, DNA ploidy
Ragnarsson-Olding *et al* (7)	2004	17	Vulva	Molecular analysis	N/A	N/A	p53 protein levels not relevant
Harting and Kim (50)	2004	11	Vulva	Retrospective series	59	10	Chemotherapy
Wechter *et al* (51)	2004	21	Vulva	Retrospective series	58	N/A	Sentinel lymphadenectomy
Edwards *et al* (52)	2004	8	Vulva	Molecular analysis	N/A	N/A	BRAF mutations not relevant
Dahlgren *et al* (53)	2005	7	Vulva	Molecular analysis	N/A	N/A	HPV not relevant
Stang *et al* (54)	2005	102	Vulva	Epidemiological study	70	N/A	No change in incidence rate
Rouzier *et al* (55)	2005	N/A	N/A	Literature review	N/A	N/A	Wide local excision with tumour-free margins, sentinel lymphadenectomy
Lundberg *et al* (56)	2006	7	Vulva	Molecular analysis	N/A	N/A	HSV not relevant
Sugiyama *et al* (10)	2007	644	Vulva	Retrospective series	68	61	Age, stage, positive inguinal lymph nodes
Dhar *et al* (57)	2007	26	Vulva	Literature review	N/A	N/A	Sentinel lymphadenectomy
Giraud *et al* (58)	2008	7	Vulva	Molecular analysis	N/A	N/A	Polyomaviruses not relevant
De Simone *et al* (59)	2008	11	Vulva	Retrospective series	53	50	N/A
Hu *et al* (11)	2010	324	Vulva	Retrospective series	N/A	N/A	Ethnicity
Moan *et al* (60)	2010	N/A	Vulva	Literature review	N/A	N/A	Sun exposure not relevant
Trifiro *et al* (61)	2010	12	Vulva	Prospective study	59	N/A	Sentinel lymphadenectomy not relevant
Terlou *et al* (62)	2010	N/A	N/A	Literature review	N/A	N/A	ABCDE and punch biopsy are useful in diagnosis
Baiocchi *et al* (63)	2010	11	Vulva	Retrospective series	64.8	10	Sentinel lymphadenectomy not relevant
Ragnarsson-Olding (64)	2011	N/A	N/A	Literature review	N/A	N/A	Sun exposure not relevant
Omholt *et al* (13)	2011	23	Vulva	Molecular analysis	N/A	N/A	KIT mutations, RAF/MEK/ERK and PI3K/AKT pathways activated
Tcheung *et al* (30)	2012	85	Genitals/vulva	Retrospective series	60.5	50.7	N/A
Schoenewolf *et al* (14)	2012	16	Genitals/vulva	Retrospective series/molecular analysis	61.9	N/A	c-KIT expression and mutations; pERK
Heinzelmann-Schwarz (this study)		33	Vulva	Literature review, retrospective series, molecular analysis	67.5	N/A	At least one of these pathological features: satellitosis, in-transit metastases, dermal mitosis, LVSI; strong c-KIT expression, lateral margin >1 cm

OS, overall survival; N/A, information not available; LVSI, lymphovascular space invasion.

**Table II:** Clinicopathological and immunohistochemical patient characteristics.

No.	Age	BD	ULC	DM	SAT	ITM	LVSI	LNP	IHC_C	IHC_M	Status
1	51	10.5	Yes	Neg	No	No	No	0	0	0	Alive
2	74	1.5	Yes	Neg	No	No	No	0	1.4	0.2	Alive
3	53	1.15	No	Neg	No	No	No	N/A	N/A	N/A	Alive
4	69	5	Yes	Neg	No	No	No	0	0	0	Alive
5	84	3.5	Yes	Pos	Yes	No	No	0	2.2	2.75	Alive
6	46	1	Yes	Neg	No	No	No	0	N/A	N/A	Alive
7	62	3.1	Yes	Neg	No	No	No	0	1	1	Alive
8	60	4	Yes	Neg	Yes	No	No	0	0.27	1.5	Deceased
9	68	4.2	Yes	Pos	No	No	No	0	0	0	Deceased
10	43	14	Yes	Pos	No	No	No	1	N/A	N/A	Alive
11	96	6	Yes	Pos	No	No	No	0	2.25	1.89	Deceased
12	91	7.5	Yes	Pos	No	No	No	0	2.11	2.11	Deceased
13	83	0	No	N/A	No	Yes	No	0	0.5	0.75	Deceased
14	84	N/A	N/A	N/A	N/A	N/A	N/A	3	N/A	N/A	Deceased
15	44	6	Yes	Pos	No	No	No	N/A	0.9	0.86	Deceased
16	71	N/A	N/A	N/A	N/A	N/A	N/A	0	N/A	N/A	Deceased
17	68	3.3	Yes	Neg	No	No	Yes	0	1.67	2.13	Deceased
18	76	5.2	Yes	Neg	No	Yes	No	0	0	0	Deceased
19	50	11	Yes	N/A	No	No	No	6	1	1.18	Alive
20	89	19.5	Yes	Pos	No	Yes	Yes	0	N/A	N/A	Alive
21	73	1	No	Neg	No	No	No	N/A	1.5	2	Alive
22	64	28	Yes	Pos	No	No	Yes	0	0	0	Deceased
23	68	7	Yes	Pos	N/A	N/A	N/A	N/A	0.75	0	Deceased
24	82	7	N/A	Pos	N/A	N/A	N/A	N/A	1	0	Deceased
25	67	N/A	N/A	N/A	N/A	N/A	N/A	0	0	0	Deceased
26	70	10	Yes	Pos	No	No	No	N/A	1	1.78	Alive
27	80	3.2	Yes	Pos	No	No	No	N/A	1.75	2.38	Alive
28	67	1	No	Neg	No	No	No	N/A	1	0.8	N/A
29	94	1.7	Yes	Neg	No	No	No	N/A	1.83	2.17	Deceased
30	34	N/A	N/A	N/A	N/A	N/A	N/A	N/A	1.09	1.36	Deceased
31	81	N/A	No	N/A	N/A	N/A	N/A	0	2.08	0.89	Alive
32	58	10	Yes	Pos	Yes	N/A	No	0	1.42	2	Alive
33	48	2	Yes	Pos	No	N/A	No	1	1.6	2.44	Alive

No., number of patients; BD, Breslow’s depth (mm); ULC, ulceration; DM, dermal mitoses </≥5 per mm^2^; SAT, satellitosis; ITM, in-transit metastases; LVSI, lymphovascular space invasion; LNP, positive lymph nodes; IHC c-KIT expression intensity (C, cytoplasmic; M, membrane); Neg, negative; Pos, positive; N/A, information not available.

**Table III:** Studies identified in the literature documenting synchronous lichen sclerosus and vulvar melanoma.

Author/(Refs.)	Year	Age	Depth (mm)	Lymph nodes	Follow-up (months)	Status
Friedman *et al* (16)	1984	14	0.7	Negative	12	NED
Egan *et al* (17)	1997	9	*In situ*	N/A	N/A	N/A
Egan *et al* (17)	1997	11	0.47	N/A	N/A	N/A
Carlson *et al* (18)	2002	83	2.7	Negative	21	NED
Hassanein *et al* (19)	2004	10	0.44	Negative	12	NED
Rosamilia *et al* (20)	2006	10	1	Positive	32	NED
De Simone *et al* (59)	2008	N/A	N/A	N/A	N/A	N/A
This study		69	1	Negative	120	DOD
This study		84	3.5	Negative	12	DOD
This study		81		Negative	2	NED

NED, no evidence of disease; N/A, information not available; DOD, death due to disease.

**Table IV:** Multivariable analysis of high-risk features.

A, Relapse-free survival			

Predictor	HR (95% CI)	aHR^a^ (95% CI)	aHR^b^ (95% CI)
Pathological characteristics	5.02 (0.62–40.61)	4.86 (0.58–40.81)	2.89 (0.35–23.83)
Lymphadenectomy	0.38 (0.12–1.15)	0.15 (0.03–0.64)	
Cell type	0.75 (0.26–2.19)	0.72 (0.23–2.22)	
Lateral margin	1.95 (0.53–7.22)	1.86 (0.49–7.03)	
c-KIT expression	2.45 (0.66–9.08)	3.13 (0.78–12.58)	2.51 (0.61–10.36)
Breslow’s depth	1.08 (1.00–1.17)		1.12 (1.02–1.22)

B, Disease-free survival			

Predictor	HR (95% CI)	aHR^a^ (95% CI)	aHR^b^ (95% CI)

Pathological characteristics			
Lymphadenectomy	0.71 (0.19–2.63)	0.25 (0.06–0.99)	0.31 (0.04–2.44)
Cell type	0.82 (0.25–2.73)	0.80 (0.24–2.72)	
Lateral margin	2.72 (0.58–12.88)	2.67 (0.56–12.76)	7.32 (0.77–69.86)
c-KIT expression	1.82 (0.38–8.67)	1.75 (0.35–8.63)	3.34 (0.42–26.29)
Breslow’s depth	1.01 (0.92–1.10)	0.93 (0.57–1.50)	

a Adjusted for Breslow’s depth;

b Final multivariate model.

aHR, adjusted hazard ratio; CI, confidence interval.
